# People serve themselves larger portions before a social meal

**DOI:** 10.1038/s41598-021-90559-y

**Published:** 2021-05-26

**Authors:** Helen K. Ruddock, Emma V. Long, Jeffrey M. Brunstrom, Lenny R. Vartanian, Suzanne Higgs

**Affiliations:** 1 School of Psychology, University of Birmingham, Birmingham, UK; 2 School of Psychology, UNSW, Sydney, Australia; 3 Nutrition and Behaviour Unit, School of Psychological Science, University of Bristol, Bristol, UK

**Keywords:** Psychology, Human behaviour

## Abstract

One of the most powerful influences on food intake yet identified is the presence of familiar others at an eating occasion: people eat much more when they eat with friends/family than when they eat alone. But why this is the case is unclear. Across two studies (Study 1: N = 98; Study 2: N = 120), we found that the mere anticipation of social interaction is all that is needed to promote the selection of larger meals, and that this occurs even when a person is alone when they make their decision. Adult women served themselves larger portions when they knew they were going to eat socially versus when they knew they were going to eat alone. These data suggest that how other people influence our food intake reaches beyond the specific eating context to affect pre-meal portion size decisions, suggesting that a fundamental shift is required in our thinking about social influences on eating.

## Introduction

Overeating is a major public health concern, negatively affecting people’s health and contributing to weight gain and obesity. Although a number of factors contribute to overeating, research has increasingly shown that environmental factors play a key role in driving excess consumption. One phenomenon that has received particular attention is the “portion size effect”—the highly reproducible tendency for people to consume more food when they are presented with a large portion^1^. Based on this research, reducing portion sizes has been advocated as a strategy to help people avoid overeating^2,3^.

Although portion size has received a great deal of attention, it is remarkable that an even more powerful effect—social facilitation of eating^4,5^—is generally overlooked. Social facilitation refers to the robust tendency for people to eat more food when dining with friends and family than when dining alone (e.g.,^4,6–8^). Social facilitation occurs in small groups and large groups, and is not limited to social occasions that coincide with other factors that influence food intake, such as alcohol consumption or eating at a celebratory event^9,10^.

Despite strong evidence for social facilitation effects on eating, surprisingly little is known about the underlying processes. The prevailing explanation is that the presence of friends or family extends the duration of a meal and, given that the predominant response to the presence of food is to eat, a longer meal results in facilitation of intake^11^. Refinements of this “time extension hypothesis” have also considered the effects of social eating on other variables that are known to influence meal size, such as not paying attention to internal signals^12^ and eating rate^13^. However, all of these explanations require the presence of additional food at social meals, which raises questions about where the additional food comes from.

Research on the control of meal size suggests that portion sizes tend to be determined and/or self-selected before a meal begins, and that most meals are consumed in their entirety^14,15^. Bridging these two areas of research, we propose a radical alternative account of socially facilitated eating. Since the earliest report of this phenomenon (e.g.,^4^), researchers have assumed that the primary stimulus is the physical presence of co-eaters. However, previous accounts of social facilitation of eating overlook the fact that, in order to consume more during social meals, it is likely that people eating socially provide themselves with more food *before* the meal than do those eating alone^16^. It is therefore possible that only the mere anticipation of social interaction is needed in order to promote the selection of larger meals. Building upon this suggestion, we propose that the selection of larger meals prior to a social occasion may occur even when a person is alone when they execute their decision. This proposal challenges long-standing assumptions about social facilitation and, in so doing, has the potential to inform the development of dietary interventions that leverage this powerful influence on food intake (e.g., portion-control strategies/advice for families that routinely eat together).

In these studies, we examined whether people make more food available for consumption when they know they will be eating with someone else versus when they know they will be eating alone. In Study 1, we examined the amount of food that participants served themselves and ate when eating alone and when eating with a friend. Participants were asked to serve an individual portion (that they themselves would like to eat) and this was done while they were alone. We also included a condition in which each participant was provided with a pot of food from which they could serve themselves throughout the meal, which is akin to previous studies of the social facilitation of eating. Thus, half of participants served themselves from a pot before eating alone/with a friend (“serve-before” condition); for the other half of participants, the pot was placed on the dining table and so participants were able to serve themselves throughout the meal (“serve-during” condition). We hypothesized that participants would serve themselves larger portions (and eat more) when eating with a friend, relative to when eating alone, and that this effect would be observed both when participants served themselves food *before* and *during* the meal. Study 2 was a conceptual replication and extension of Study 1. Previous research indicates that social facilitation is stronger when people eat with people who are familiar to them^5^. To determine whether this extends to self-serving before a meal, we explored whether participants choose larger portions before dining with a friend than with a stranger, and whether these portions are larger than those selected before dining alone.

## Study 1: Methods

### Participants

Ninety-eight female participants (*N* = 49 pairs of friends) took part in this study. Findings from previous research suggest that gender may moderate effects of social influences on eating behavior (e.g.,^5^) and so we decided to recruit females only. The sample size was calculated to provide 80% power to detect medium-sized effects and interactions (*f* = 0.25). To disguise the aims of the study, participants were led to believe that the purpose was to examine people’s problem-solving abilities when working alone and with a friend. Ethical approval was obtained from the University of Birmingham’s ethics committee. The study adhered to relevant regulations for conducting human behavioral research, and all participants gave written informed consent before taking part. The study method and data analysis plan were preregistered on the Open Science Framework website (https://osf.io/6xf5v).

### Materials and measures

#### Food

Each participant was provided with a pot of pasta from which they could serve themselves lunch. This consisted of Tesco Conchiglie pasta mixed with 350 g Tesco Tomato & Basil Sauce and 50 g Tesco British Medium Cheddar Cheese. The total (cooked) weight of food available in each serving pot was 1030 g.

#### Appetite and liking ratings

Assessments of hunger, fullness, and “liking” for the lunch meal were taken using 100-mm Visual Analogue Scales (VAS), anchored by *Not at all* on the left and *Extremely* on the right. A composite “appetite” score was calculated by taking the mean rating assigned to the “hunger” VAS and the inverse rating assigned to the “fullness” VAS (100-fullness). The liking VAS was anchored by *Didn’t like it at all* and *Liked it a lot* to the left and right of the scale, respectively.

### Procedure

Participants attended two sessions, separated by at least seven days. In one session, the participant attended alone and in the other session the participant attended with a friend. The order in which participants completed the “social” and “alone” conditions was counterbalanced. Participants were randomly allocated to either the “serve-before” or “serve-during” condition. All sessions took place between 12:00 pm and 2:00 pm and participants were instructed to refrain from eating for at least three hours prior to the session.

Upon arrival, participants were seated in individual waiting rooms and completed ratings of hunger and fullness. Next, participants served themselves lunch. Participants in the serve-before condition were invited to serve themselves as much pasta as they wished from a pot, prior to the meal. When they were in the social condition, the participant and friend served themselves individually from separate pots, and so participants were unaware of how much their friend had served themselves. Hence, we were able to compare the same serving activity across social and non-social conditions and ensure that the serving decision was not influenced by how much their friend had served themselves. Participants then took their selected portion into a dining room and were instructed to eat as much as they wanted (participants in the social condition ate in the same room as their friend).

For participants in the serve-during condition, the serving pots were placed in the dining room so that participants could serve themselves throughout the meal. As in the serve-before condition, each participant was provided with their own separate pot of pasta from which to serve themselves. Participants were left alone to serve themselves and eat as much as they liked.

In both serving conditions, participants informed the experimenter when they were finished eating and the duration of the meal was covertly timed by the experimenter. Serving dishes were covertly weighed before and after the meal to determine the amount of food that participants had served themselves. Following the meal, participants completed VAS measures of hunger, fullness, and liking for the lunch meal. They were then given 10 min to solve a list of anagrams (in line with the cover story) and afterwards were taken into individual testing rooms in which they completed additional measures (see supplementary materials). The experimenter then measured the participant’s height and weight and participants were fully debriefed.

### Data analysis

Outlying values were identified using Hoaglin and Iglewicz's^17^ outlier labeling rule. One outlier was identified but removal of this data point did not change the findings and so we report analyses with this participant *included* in the dataset. Because data were non-independent (i.e., participants signed up to the study in pairs), a multilevel model (MLM) was conducted to examine the effect of social context condition and serving condition and their interaction on the amount of food served. Condition order (i.e., alone first vs. social first) was included as a fixed factor. Variables that correlated with the amount of food served were included as covariates. Exploratory analyses in line with the preregistration plan were conducted and are reported in the supplementary materials.

## Study 1: results

### Preliminary analyses

Fifty participants (25 pairs) were allocated to the serve-before condition, and 48 participants (24 pairs) to the serve-during condition. Participant characteristics, stratified by serving condition, are presented in Table 1. Across serving conditions, participants did not differ on any of the examined characteristics, *F*(9, 84) = 0.93, *p* = 0.508, Wilk’s Λ = 0.91. Twelve participants guessed the aims of the study. Removal of these participants did not change the overall findings and so they were retained in the analyses.

**Table 1:** Participant characteristics stratified by serving condition. Values are means (standard deviation).

Variables	Serve-before (*n* = 50)	Serve-during (*n* = 48)	Univariate test statistic
BMI (kg/m^2^)	24.08 (3.32)	22.89 (3.35)	*F*(1, 92) = 3.11, *p* = .081
Age (years)	20.12 (2.77)	20.68 (5.96)	*F*(1, 92) = 0.36, *p* = .552
Food liking^†^	76.32 (13.76)	72.35 (15.12)	*F*(1, 92) = 1.77, *p* = .186
Pre-meal appetite ratings^†^	72.83 (13.28)	70.79 (14.17)	*F*(1, 92) = 0.52, *p* = .475

^†^Mean scores collapsed across social and alone conditions.

Participants consumed over 90% of the food that they served themselves in all conditions (Serve-before/Alone: *M* = 94.07%, *SD* = 10.55; Serve-before/Social: *M* = 95.31%, *SD* = 7.70; Serve-during/Alone: *M* = 92.90%, *SD* = 12.39; Serve-during/Social: *M* = 93.33%, *SD* = 10.49). Due to the high concordance between the amount served and eaten, we only report results for amount *served* because this is most central to our novel hypothesis (analyses of amount eaten yielded similar findings). Hunger ratings prior to the meal correlated with the amount of food participants served themselves (*r* = 0.23, *p* = 0.001) and so this was included as a covariate.

### Main analysis: effect of social context and serving condition on amount served

**Figure 1:**
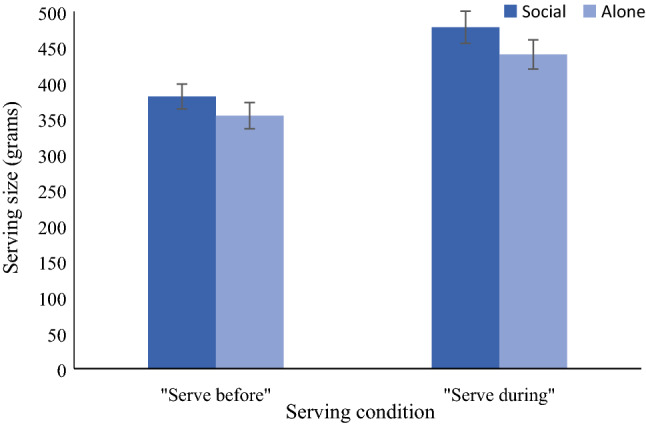
Amount served (grams) as a function of serving condition and social context condition. Values are means, and error bars represent the standard error of the mean.

Participants served themselves larger portions when they ate with their friend, relative to when they ate alone, *F*(1, 97.47) = 4.77, *p* = 0.031, 95% CI [2.46, 51.74], Cohen’s *d* = 0.26 (Fig. 1). Participants in the serve-during condition also served themselves significantly larger portions than did those in the serve-before condition, *F*(1, 98.19) = 15.92, *p* < 0.001, 95% CI [47.75, 142.24], Cohen’s *d* = 0.67 (Fig. 1). There was no interaction between serving condition and social context condition on amount served, *F*(1, 92.80) = 0.38, *p* = 0.537.

## Study 2

The results from Study 1 supported our hypothesis that participants would serve more food for themselves when eating with a friend compared with when eating alone. We also found, for the first time, that this was the case when they served themselves before the meal and when they served themselves during the meal. The aim of Study 2 was to replicate the finding that participants served themselves more food before the meal. Because there is evidence to suggest that social facilitation of eating is less likely to be observed when eating with strangers^5^, we extended the study to examine the effect of both familiar and unfamiliar companions on portions served before the meal. We hypothesized that participants in the “friend” condition would serve themselves larger portions than those in the “alone” condition. Given the lack of published data, we made no specific prediction about whether people would serve themselves more when eating with strangers versus eating alone.

## Study 2: methods

### Participants

Participants were 120 women recruited from the undergraduate psychology cohort at UNSW Sydney and from the community (an additional 80 people were also recruited as eating companions for the social conditions). The sample size was calculated to provide 80% power to detect small-to-medium effects. Six participants were excluded for the following reasons: in a “stranger” condition session, the participant reported that they were friends with their eating companion but the companion reported that they were strangers (*n* = 4); a participant reported sharing their food with their eating companion (*n* = 1); and a participant was eating their own food from their bag when the experimenter entered the room at the conclusion of the lunch task (*n* = 1). The final sample consisted of 114 participants. This study was approved by the UNSW ethics committee, and all methods adhered to relevant regulations for conducting human behavioral research. All participants gave written informed consent prior to taking part. The design and analyses of this study were preregistered with AsPredicted (https://aspredicted.org/9ab3q.pdf).

### Materials and measures

#### Food

Participants individually served themselves lunch from a large serving pot. In the alone condition, the lunch comprised 365 g of Woolworths macaroni pasta, combined with 240 g of Leggo’s tomato, onion, and garlic sauce (total 605 g). In the friend and stranger conditions, twice this amount was provided in a single serving pot (total 1210 g). Previous research has found that female undergraduate students consumed approximately 276 g of pasta, on average (e.g.,^18,19^). For the present study, to ensure that participants would not be restricted by how much pasta was available, a portion roughly double that amount was provided per participant.

#### Questionnaires

The following measures formed an electronic questionnaire pack, which participants completed on a tablet at the end of the eating component of the study:

*Recent food intake.* Participants indicated the time at which they ate their last meal (i.e., the meal prior to their study session commencing). This value was used as a proxy for participants’ baseline level of hunger.

*Liking of the pasta.* To assess liking for the food provided, participants were asked “How much did you like the pasta?” Participants responded by moving a marker on a sliding scale ranging from 0 to 100.

*Demographics.* Participants reported their gender, age (in years), height (in centimeters), weight (in kilograms), and ethnicity. Each participant’s height and weight was used to calculate their BMI.

*Additional questionnaires.* For exploratory analyses, several additional measures were included to examine potential mediators and moderators of the social facilitation effect. A summary is provided in the supplementary material.

### Procedure

Participants signed up for a study investigating the way in which “impressions and opinions form after viewing media presentations.” Because the friend and stranger conditions required participants to eat with another person, and this pairing needed to be organized in advance, timeslots were randomly allocated to one of the three social contexts. Alone timeslots only allowed one person to sign up; stranger timeslots allowed two people to sign up; and friend timeslots allowed one person to sign up, who was then contacted and asked to bring a same-gender friend to the study session. As in Study 1, participants were asked to refrain from eating for three hours prior to their scheduled session. Study sessions were scheduled to begin between 11:00 am and 3:00 pm.

Participants came to the laboratory dining room either alone or in pairs, depending on their assigned condition. The first person to sit at the dining table was determined to be the main participant; the second person (friend and stranger conditions only) was determined to be the eating companion. The distinction between main participant and eating companion was made for the purposes of the serving procedure and data collection, as well as for data analysis; participants were unaware that this distinction existed. All participants were told that the researchers were interested in the way that impressions and opinions form after watching media presentations.

Participants watched 10 min of a “60 Minutes Australia” news segment. At the conclusion of the video, participants were told that they would be having pasta for lunch. As in Study 1 (serve-before condition), participants in the social conditions took turns to serve themselves pasta in the laboratory kitchen. While one person served themselves lunch the other person remained in the dining room and wrote down some discussion questions about the video. The main participant served themselves first so that the amount of food available in the serving pot was consistent for all main participants. A plate cover was put over the main participant’s bowl once they had served themselves so that the friend/stranger would not be able to see how much food had been served. Participants were told that they would be having a discussion about the video while having their lunch.

In the alone condition, participants served themselves in the kitchen and then returned to the dining room. They were asked to imagine that they were going to have a conversation with someone about the video that they had just watched, and wrote down discussion questions they would ask in this imagined interaction.

In all conditions, the experimenter covertly weighed each person’s individual bowl of pasta to determine the amount of food served, before bringing the food and a jug of water into the dining room on a trolley. Participants also had access to a communal container of breadsticks that the researcher placed on the dining table. The consumption of breadsticks was measured by self-report but these reports were found to be unreliable and analyses involving breadstick consumption are therefore not reported. Participants were informed that they would be given 20 min to complete the task, although they were free to let the experimenter know that they had finished if they were done before the experimenter returned to the dining room. The time-limit for the meal was based on findings from Study 1 in which participants ate for 11.5 min and 16.5 min, on average, in the alone and social conditions respectively. The experimenter timed how long the participants took to complete the lunch task.

After finishing the lunch task, participants completed the questionnaire pack. While participants completed the questionnaires, their bowls were weighed again to determine the amount of pasta consumed. After the questionnaires were completed, participants were debriefed.

### Data analysis

As in Study 1, data were screened for outliers. There was one outlier but, given that removing that participant did not impact the pattern of results, the full sample was included in the following analyses. To test whether social effects would be observed among participants eating with friends and strangers, a planned-contrasts (friend vs. alone; stranger vs. alone) analysis approach was used to examine the effect of social context on the amount of pasta served.

The analyses were conducted on the main participants’ data only (participants in the alone condition did not participate in the study with a partner, and so it was not appropriate to conduct an MLM to compare effects of condition on food served by *both* participants in the friend and stranger conditions). The amount of pasta served did not correlate with time since last food intake, age, or participants’ BMI and so no variables were included as covariates.

## Study 2: results

### Preliminary analyses

Participant characteristics, stratified by social context, are presented in Table 2. Across social contexts, participants did not differ on the examined characteristics, *F*(8, 216) = 1.19, *p* = 0.305, Wilk’s Λ = 0.92. Participants consumed over 90% of the food that they served themselves in all conditions (Alone: *M* = 97.74%, *SD* = 9.43; Friend: *M* = 94.37%, *SD* = 11.55; Stranger: *M* = 95.06%, *SD* = 15.74). Due to the high concordance between the amount served and eaten, we only report results for amount *served*, again because this is most central to our novel hypothesis (the analyses of amount eaten revealed the same effect of social context).

**Table 2:** Participant characteristics stratified by social context condition. Values are means (standard deviation).

Variables	Alone (*n* = 40)	Friend (*n* = 39)	Stranger (*n* = 35)	Univariate test statistic
BMI (kg/m^2^)	21.48 (2.66)	22.27 (3.72)	22.06 (3.50)	*F*(2, 111) = 0.60, *p* = .553
Age (years)	19.42 (2.28)	19.21 (2.02)	18.86 (1.48)	*F*(2, 111) = 0.78, *p* = .460
Food liking	74.78 (19.47)	65.00 (22.49)	67.51 (17.99)	*F*(2, 111) = 2.50, *p* = .086
Time since last food intake	6 h 55 m (5 h 21 m)	8 h 11 m (6 h 52 m)	7 h 17 m (5 h 37 m)	*F*(2, 111) = 0.47, *p* = .629

### Main analysis: effect of social context on amount served

**Figure 2:**
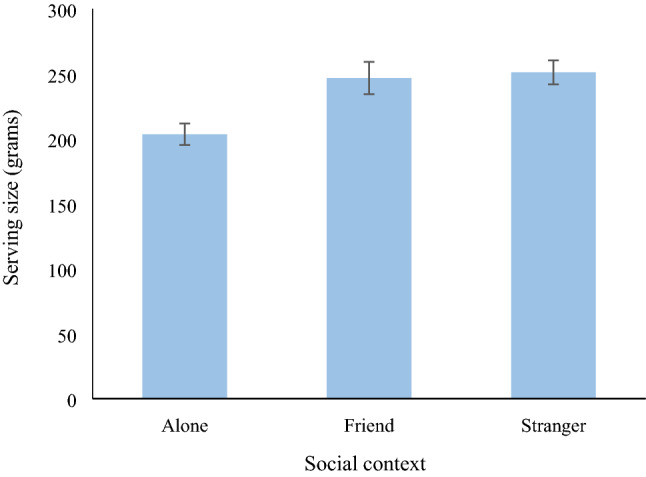
Amount served (grams) as a function of social context condition. Values are means, and error bars represent the standard error of the mean.

Participants in the friend condition served significantly more pasta (contrast estimate = 43.24, *SE* = 14.11, *p* = 0.003, 95% CI [15.29, 71.20], Cohen’s *d* = 0.65) than did participants in the alone condition. Participants in the stranger condition also served significantly more pasta (contrast estimate = 47.66, *SE* = 14.51, *p* = 0.001, 95% CI [18.91, 76.41], Cohen’s *d* = 0.89) than did participants in the alone condition (see Fig. 2).

## Discussion

Across two studies, we found that participants who knew that they were going to eat socially served themselves a larger portion before the meal than did participants who knew that they were going to be eating alone. In other words, they served themselves more food in *anticipation* of eating with others. Hence, we have demonstrated for the first time that the physical presence of co-eaters is not necessary for intake to be socially facilitated and that knowledge about the social context of eating is factored into decisions about portion size that occur prior to a meal. These results mean that a fundamentally different approach to understanding how eating socially affects intake is now required. Rather than asking why people eat more when they are in groups than when they are alone, we need to ask what causes people to select more food in the first instance.

Previous explanations for the social facilitation of eating focused primarily on factors that influence food intake during a meal and that relate to the presence of others. For example, the time extension hypothesis posits that people eat more during social meals because social meals last longer than lone meals^11^. Our results suggest an alternative causal relationship: Social meals last longer because people ensure that more food is available in advance. It has been noted previously that, for social facilitation of eating to occur, more food must be made available for consumption^12,16^, which has been referred to as social “precilitation” of intake. We have shown that one way in which more food can materialize is by people selecting larger portions in advance of social meals. Hence, the social facilitation of eating may differ from other forms of behavioral facilitation that relate specifically to the activating effects of presence of others on a predominant behavioral response.

The fact that people adjust portion sizing according to whether a meal will be eaten socially suggests that social facilitation of eating may be underpinned by a previously unrecognized form of dietary learning, which we refer to as anticipated social facilitation. Pre-meal planning is thought to reflect prior learning about the consequences of consuming foods (e.g., how tasty and filling they are)^20^. Social context may therefore influence pre-meal decisions because people have learned that the consequences of eating socially are different from the consequences of eating alone, which motivates larger portion-size selections. Specifically, it is possible that people learn that a particular food tastes better or is less satiating in a social context and they then draw on this experience to adjust the amount that they self-serve when they encounter the food at subsequent social occasions. Indeed there is some support for the idea that food is perceived as more pleasant tasting when the consumption experience is shared compared to when the experience is unshared^21^, though this has not been consistently demonstrated (e.g.,^22^). Furthermore, the idea that meals eaten socially are less satiating than meals eaten alone is tentatively supported by findings from one study in which participants reported a similar decline in hunger ratings, even though those who dined socially consumed significantly more than did those who ate alone^23^. Further research is clearly needed to establish the effects of social context on appetite and food pleasantness perceptions, and to examine the suggestion that social context modulates expectations about how tasty and filling a food will be via learning.

In Study 2, we observed that participants served more food and then ate more food when eating socially than when eating alone, and this was the case for both familiar and unfamiliar dining companions. Previous evidence suggests that the social facilitation of eating is much more likely to be observed for groups of friends and family than for groups of strangers^5^. This might be because people tend to eat less in the presence of people they do not know to avoid being evaluated negatively^24^. It is possible that such impression management concerns have a greater effect on amounts consumed *during* a meal than on amounts served *prior* to a meal. However, the fact that participants ate all of the food that they served themselves in the stranger condition suggests that their intake during the meal was not inhibited by impression management concerns. One explanation for this could be that participants in the “stranger” condition were somewhat familiar with each other. Indeed, the majority of participants were students at the same university and so it is possible that they knew each other from their classes. It could be the case that in order to see a weaker/absent social facilitation effect among strangers, participants need to be *completely* unfamiliar with their eating companions.

The current findings have practical implications. We observed anticipation of social facilitation in the context of an everyday meal, which suggests that the phenomenon applies to regular family meals and is not confined to celebratory events or special occasions at which it might be expected that overeating is part of a social script. Across two studies, we found small (Study 1) to medium (Study 2) sized effects of social context on the amount of food participants served themselves. Although overeating at a regular family dining event will have a trivial impact on energy balance, over time, the effect could be significant. Our work shows that the likely origin of this key driver of overconsumption is in the kitchen before a meal begins, and future research should establish the longer-term effects of social eating on energy balance and body weight. Another important avenue for future research is to establish the extent to which people are aware of their decision to provide themselves with more food prior to a social meal. Indeed, raising awareness of anticipated social facilitation could play an important role in mitigating this effect and future studies should consider the impact of such an intervention. It may also be advisable, for those who wish to avoid overeating in social situations, to pay attention to the amount of food that will be available at such occasions rather than employing strategies to avoid consuming food when at the table. Notably, in Study 1, participants who served themselves *before* the meal selected smaller portions than did those who were able to serve themselves *during* the meal. Combining such pre-meal planning with an awareness of social facilitation of eating may help to minimize unwanted consumption while being able to enjoy social eating occasions. In a single meal the effects will be trivial. Over time, however, this simple strategy could play an important role in promoting healthy weight maintenance.

To conclude, we provide novel insight into how social context influences food intake: Our findings suggest that people eat more during social meals *because* they make more food available. Therefore, to understand why people eat more in social situations, future research should focus on establishing how social factors impact upon *pre-meal* decisions about what and how much to eat.

## Supplementary Information

Supplementary Information.

## Data Availability

The datasets generated during the current research will be made available via the UK Data Service repository upon publication.
