# Supplementary material for: People serve themselves larger portions before a social meal
Source: Sci Rep. 2021 May 26;11:11072. doi: 10.1038/s41598-021-90559-y (PMC8155033; doi:10.1038/s41598-021-90559-y)
Supplement: Supplementary file 1 — Supplementary Information. [file 41598_2021_90559_MOESM1_ESM.docx]

People serve themselves larger portions before a social meal

(supplementary material)

Helen K. Ruddock^1^, Emma V. Long^2^, Jeffrey M. Brunstrom^3^, Lenny R. Vartanian^2^, *Suzanne Higgs^1^

^1^ School of Psychology, University of Birmingham, Birmingham, United Kingdom

^2^ School of Psychology, University of New South Wales, Sydney, Australia

^3^ Nutrition and Behaviour Unit, School of Psychological Science, University of Bristol, Bristol, United Kingdom

***Corresponding author**: Professor Suzanne Higgs. **E-Mail:** [s.higgs.1@bham.ac.uk](mailto:s.higgs.1@bham.ac.uk)

# Study 1: Supplementary Methods

## Questionnaires

### *Three-Factor Eating Questionnaire-18*

The Three-Factor Eating Questionnaire Revised 18-item version (TFEQ-18) was included to assess dietary behavior ^1^. The instrument is a shortened and revised version of the original 51-item TFEQ ^2^. The questionnaire measures dietary restraint (i.e., attempts to restrict food intake in order to control body weight), uncontrolled eating (i.e., tendency to experience a loss of control over eating), and emotional eating (i.e., eating in response to negative moods). Responses for each item are provided on a 4-point scale (e.g., definitely true/mostly true/mostly false/definitely false), and assigned a score between 1 and 4. Item scores are summated to provide a score for each of the three subscales. Cronbach’s alpha indicated good internal reliability for each subscale (dietary restraint: α = .79; uncontrolled eating: α = .80; emotional eating: α = .81).

### *Attribution questionnaire*

To examine whether participants were aware of any influence that social factors had on their serving selection, they rated the extent to which a variety of factors had influenced their serving-size selection (as in ^3^). Ratings were provided on a 9-point scale ranging from -4 (*made me eat less than I normally would*) to +4 (*made me eat more than I normally would*). For the purposes of this study, we were interested in the rating that participants assigned to the item “the presence of my friend.” Additional filler items (e.g., “how hungry I was”) were included to disguise the aim of the study. Participants who indicated that the presence of their friend had influenced how much they ate were asked to write down why they thought this was the case.

## Procedure (additional information)

At the end of Session 2, participants were taken into individual testing rooms in which they completed the following measures: (1) demand awareness (participants were asked to write down what they thought were the aims of the study), (2) friend familiarity (participants stated how long they had known their friend, and rated on 10-point scales how well they know their friend and how close they feel to their friend [*Not very* and *Very* were assigned to values 1 and 10, respectively]), (3) demographics (age and ethnicity), (4) attribution questionnaire, and (5) TFEQ-18.

# Study 1: Supplementary Results

## Effect of condition on food liking ratings

We explored whether liking ratings differed as a function of social context and serving condition. Liking ratings were entered into an MLM with social context condition, serving condition, and condition order as fixed factors. There was no main effect of social context condition, *F*(1, 97.49) = 2.52, *p* = .116 (social condition: *M* = 75.72, *SD* = 15.44; alone condition: *M* = 73.68, *SD* = 16.21), and no main effect of serving condition, *F*(1, 98.16) = 1.53, *p* = .219, on liking ratings (serve-before: *M* = 76.45, *SD* = 15.07; serve-during: *M* = 72.91, *SD* = 16.45). There was also no significant social context × serving condition interaction on liking ratings, *F*(1, 97.49) = 3.21, *p* = .076.

## Effect of condition on pre-meal appetite

MLM analysis was conducted to explore whether participants’ appetite differed as a function of condition. Composite pre-meal appetite scores were calculated as the mean of hunger and the inverse of fullness (100 minus fullness). Social context condition, serving condition, and condition order were entered as fixed factor predictors of pre-meal appetite. There was no main effect of social context condition on pre-meal appetite ratings, *F*(1, 98.03) = 1.80, *p* = .183 (social condition: *M* = 73.01, *SD* = 16.10; alone condition: *M* = 70.05, *SD* = 18.65). There was also no main effect of serving condition, *F*(1, 98.40) = 0.88, *p* = .350, (serve-before: *M* = 72.82, *SD* = 17.24; serve-during: *M* = 70.21, *SD* = 17.61), and no social context condition × serving condition interaction on pre-meal appetite ratings, *F*(1, 98.02) = 0.21, *p* = .651.

## Effect of condition on meal duration

Meal duration (secs) was entered as the dependent variable in an MLM with social context, serving condition, and condition order as fixed factors. Pre-meal appetite ratings correlated positively with meal duration, *r* = .16, *p* = .024, and so pre-meal appetite was included as a covariate. There was a main effect of social context condition, *F*(1, 97.07) = 73.94, *p* < .001, and serving condition, *F*(1, 98.25) = 12.71, *p* = .001, on meal duration. Meal duration was significantly longer in the social condition (*M* = 988.38, *SD* = 393.07) relative to alone condition (*M* = 686.08, *SD* = 358.75), and in the serve-during condition (*M* = 946.91, *SD* = 432.98) relative to the serve-before condition (*M* = 729.35, *SD* = 344.85). There was also a social context condition × serving condition interaction on meal duration, *F*(1, 96.40) = 6.20, *p* = .014. Pairwise comparisons revealed that, when participants ate socially, meal duration was significantly longer in the serve-during condition (*M* = 1139.30, *SD* = 385.39) relative to the serve-before condition (*M* = 840.54, *SD* = 344.23), *p* < .001. Meal duration did not differ significantly between serving conditions when participants ate alone (serve-before: *M* = 620.38, *SD* = 311.91; serve-during: *M* = 754.53, *SD* = 393.45; *p* = .051).

## Food intake attributions

Table S1 shows the frequency (%) by which each attribution was assigned a score of < -1 (“made me eat less”), 0 (“made me eat the same”), and >1 (“made me eat more”). Of particular interest was the attribution assigned to the ‘friend’s presence’ attribution, in which less than half (40%) of participants thought that the presence of their friend made them eat more.

# Study 2: Supplementary Methods

## Additional questionnaires

### Attention to food intake

All participants were asked “On a scale from 0 to 100, how much attention were you paying to the amount of pasta you were eating?” Participants in the “friend” and “stranger” conditions were also asked “How much attention were you paying to the amount of pasta the other person was eating?”.

### Pleasantness of the dining experience

Participants were asked “On a scale from 0 to 100, how pleasant was the dining experience?”

### *Perceived appropriateness*

Participants were asked “On a scale from 0 to 100, how appropriate was the amount of pasta that you ate?”

### Impression management concerns

Participants in the “friend” and “stranger” conditions were asked “On a scale from 0 to 100, how concerned were you with what the other person thought about the amount of pasta you were eating?” and “On a scale from 0 to 100, how concerned were you about the impression that the other person had of you?” Given that responses to these two questions were correlated (*r* = .52, *p* < .001), these two items were averaged to create an index of impression management concerns.

### Self-monitoring

Snyder’s ^4^ Self-Monitoring Scale was used to assess self-monitoring orientation. The scale contains 25 items that investigate the ways in which individuals monitor and adjust their behavior in response to social cues that indicate what is appropriate in a given situation. Items were scored in the direction of high self-monitoring and then summed, with higher scores indicating greater self-monitoring orientation. Cronbach’s α for the present study was .69.

Dietary restraint

Herman and Polivy’s ^5^ Revised Restraint Scale was used to assess participants’ levels of dietary restraint. This scale is a 10-item measure investigating eating behaviors, intentions to diet, and weight fluctuations. Scores across the 10 items were summed to give a total score, with higher total scores indicating a higher level of dietary restraint. In the present study, Cronbach’s α was .81.

# Study 2: Supplementary Results

## Moderation analyses

Exploratory analyses were conducted to examine whether the effects of condition on amount served were moderated by scores on the Dietary Restraint Scale, or Self-Monitoring Scale. Two-way ANOVAs were conducted, with condition as the independent variable, amount served/eaten as the dependent variables, and scores on the Dietary Restraint Scale or Self-Monitoring Scale as moderators. There were no significant interactions between eating condition and either self-monitoring (all *p*s > .103) or dietary restraint (all *p*s > .410) on the amount of food served or eaten.

## Possible mechanisms for the social facilitation effect

Measures of “perceived appropriateness,” “attention to food intake,” and “impression management concerns” were analysed to examine the extent to which they underlie effects of condition on amount served/eaten. Mediation analyses were conducted on the main participants’ data only. Exploring potential mediators involved two steps. First, one-way ANOVAs were conducted on the potential mediators (attention to own food intake; attention to the eating companion’s food intake [friend and stranger conditions only]; perceived appropriateness of own food intake; and impression management concerns [friend and stranger conditions only]) to establish whether there were any between-group differences, and hence whether mediation analyses would be appropriate. Any potential mediator that differed significantly between groups was then included in a mediation analysis, using PROCESS (Model 4; ^6^), to examine whether that variable mediated the effect of eating condition on the primary outcome variables. PROCESS constructs percentile bootstrap confidence intervals for the indirect effect using a random resampling with replacement method. In the present study, a confidence level of 95% was used and the confidence intervals that were generated were based off 5,000 bootstrap samples.

There were no significant differences between social context conditions for three of the potential mediators of the social facilitation effect: attention to own food intake (*F*(2, 111) = 0.81, *p* = .448, η_p_^2^ = 0.01), attention to eating companion’s food intake (*F*(1, 72) = 0.06, *p* = .811, η_p_^2^ = 0.001), and perceived appropriateness of own food intake (*F*(2, 111) = 2.94, *p* = .057, η_p_^2^ = 0.05). See Table S2 for mean values on each of these variables stratified by condition.

### Impression management

There were significant differences in impression management concerns between conditions (*F*(1, 72) = 7.61, *p* = .007, η_p_^2^ = 0.10), such that participants in the friend condition (*M* = 25.94, *SD* = 22.46) were significantly less concerned about the impression that their eating companion had of them than were participants in the stranger condition (*M* = 41.44, *SD* = 25.90). However, mediation analyses showed no significant indirect effect of condition through impression management concerns on either the amount of pasta served (bootstrap 95% CI [-8.41, 19.09]) or the amount of pasta eaten (bootstrap 95% CI [-13.61, 8.92]).

## Multilevel Modelling Analysis of Amount Served and Eaten

Because data were also collected from the eating companion in the friend and stranger conditions, supplementary analyses were conducted that included both members of the dyad to confirm the pattern of results in the social context conditions. These data were nested in structure (i.e., the data of each participant and their eating companion were nested within a particular study session), and therefore multilevel modeling was used to control for the interdependencies in the data. The primary outcome variables were included at Level-1 and the dyad ID was included at Level-2. Models were run with full maximum likelihood estimation. Continuous predictor variables (amount of pasta served by the eating companion, and amount of pasta eaten by the companion) were centered around the grand mean, and eating condition was dummy-coded (0 = friend, 1 = stranger). Because there were more than 50 groups (but less than 100 groups) in the sample, results were reported with asymptotic standard errors ^7^. Given that individuals in the alone condition did not participate in the study with another person, their data were not included in the multilevel models.

Social context (friend vs. stranger) was not a significant predictor of the amount of pasta served by the main participants (*b* = 6.39, *SE* = 12.00, *t*(72) = 0.53, *p* = .596). Although the amount of pasta served by eating companions was a significant predictor of the amount of pasta served by main participants (*b* = 0.16, *SE* = 0.08, *t*(73) = 2.03, *p* = .046), this result was not of practical significance given that all participants served themselves without knowing how much food their companion had served. There was no significant interaction between the amount of pasta served by eating companions and the social context condition (*b* = -0.11, *SE* = 0.16, *t*(72) = -0.65, *p* = .519).

With respect to the amount of pasta eaten, neither social context (*b* = 7.65, *SE* = 12.97, *t*(72) = 0.59, *p* = .557) nor the amount of pasta eaten by the eating companions (*b* = 0.17, *SE* = 0.10, *t*(73) = 1.70, *p* = .093) significantly predicted the amount of pasta eaten by the main participants. There was also no significant interaction between the amount of pasta eaten by companions and social context (*b* = -0.21, *SE* = 0.20, *t*(72) = -1.04, *p* = .302).

Table S1. *Frequency (%) of responses for each attribution.*

|  | Made me eat less (-4 to -1) | | Made me eat the same (0) | | Made me eat more (1 to 4) | | |
| --- | --- | --- | --- | --- | --- | --- | --- |
| How hungry I was | | 7.22 | | 4.12 | | 88.66 |  |
| My mood | | 11.46 | | 42.71 | | 45.83 |  |
| Medical condition | | 5.21 | | 88.54 | | 6.25 |  |
| The presence of my friend | | 19.59 | | 40.21 | | 40.21 |  |
| The absence of my friend | | 30.93 | | 48.45 | | 20.62 |  |
| To get ‘energy’ for the problem solving task | | 2.06 | | 67.01 | | 30.93 |  |
| How tasty I thought the meal would be | | 9.28 | | 16.49 | | 74.23 |  |
| What was expected of me | | 2.06 | | 60.82 | | 37.11 |  |
| It was free food | | 1.03 | | 19.59 | | 79.38 |  |

*N.B. Participants were asked “Please rate whether, and to what extent, each of the factors listed below influenced how much food you served yourself”. Responses were provided on a 9-point scale ranging from -4 to 4.*

Table S2. *Potential Mediators Stratified by Social Context Condition. Values are Means (Standard Deviation).*

| Potential mediators | Alone (*n* = 40) | Friend (*n* = 39) | Stranger (*n* = 35) |
| --- | --- | --- | --- |
| Attention to own intake | 57.43 (22.48) | 53.41 (22.94) | 50.97 (21.33) |
| Attention to companion’s intake | – | 35.13 (25.87) | 36.69 (29.89) |
| Appropriateness of own intake | 85.40 (11.88) | 80.21 (15.44) | 76.57 (19.90) |
| Impression management concerns | – | 25.94 (22.46) | 41.44 (25.90) |

# SI References

1. Karlsson, J., Persson, L.-O., Sjöström, L. & Sullivan, M. Psychometric properties and factor structure of the Three-Factor Eating Questionnaire (TFEQ) in obese men and women. Results from the Swedish Obese Subjects (SOS) study. *Int. J. Obes.* **24**, 1715–1725 (2000).

2. Stunkard, A. J. & Messick, S. The Three-Factor Eating Questionnaire to Measure Dietary Restraint, Disinhibition and Hunger. *J. Psychosom. Res.* **29**, 71–83 (1985).

3. Vartanian, L. R., Reily, N. M., Spanos, S., Herman, C. P. & Polivy, J. Self-reported overeating and attributions for food intake. *Psychol. Health* **32**, 483–492 (2017).

4. Snyder, M. Self-monitoring of expressive behavior. *J. Pers. Soc. Psychol.* **30**, 526–537 (1974).

5. Herman, C. P. & Polivy, J. Restrained eating. in *Obesity* (ed. Stunkard, A. J.) 208–225 (Philadelphia, PA: Saunders, 1980).

6. Hayes, A. F. *Introduction to Mediation, Moderation, and Conditional Process Analysis*. (The Guildford Press, New York, 2017).

7. Maas, C. J. M. & Hox, J. J. Robustness issues in multilevel regression analysis. *Stat. Neerl.* **58**, 127–137 (2004).
